# Almost 200 years of anthocyanin research: What we know, what we assume and what remains unknown

**DOI:** 10.1111/plb.70283

**Published:** 2026-09-03

**Authors:** N. Choudhary, N. Khatun, B. Pucker

**Affiliations:** ^1^ Plant Biotechnology and Bioinformatics Institute for Cellular and Molecular Botany (IZMB), University of Bonn Bonn Germany

**Keywords:** Coloration, evolution, flavonoids, MYB, pigmentation, transcriptional regulation

## Abstract

Flavonoids are frequently presented as health‐beneficial plant metabolites, and their subclass anthocyanins is best known for striking flower colours. The biosynthesis of flavonoids has been studied for decades and has developed into a model system in plant biology. Despite thousands of publications about this pathway, there are still open questions about fundamental aspects of anthocyanin biosynthesis. Here, we review the evolution of anthocyanin biosynthesis and highlight key open questions–some of which are sitting in plain sight.

## Graphical abstract

Nearly 200 years of anthocyanin research reveal a well‐understood anthocyanin biosynthetic pathway. However, there are still open questions regarding its evolution, functions and ecological significance.

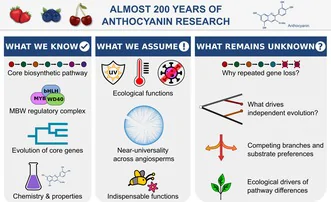

## INTRODUCTION

Few plant traits have captivated scientists for as long, or as productively, as the colour of plants. When Robert Boyle described the pH sensitivity of ‘syrup of violets’ in *Experiments and Considerations Touching Colours* (1664), and when Gregor Mendel reported the dominance of purple over white pea flowers two centuries later, neither knew they were observing what we now call anthocyanins (Boyle 1664; Mendel 1865). Boyle discovered a pigment responsive to acids and bases, while Mendel recognized a heritable trait. Only much later were these chemical and genetic observations unified (Winkel‐Shirley 2001; Hellens *et al*. 2010; Feng *et al*. 2025; Grünig *et al*. 2025). In 1835, Ludwig Clamor Marquart introduced the name ‘Anthokyan’ for the blue flower pigment of cornflower described in his treatise ‘Die Farben der Bluthen’ (Marquart 1835). As chemical nomenclature standardized during the late nineteenth century, the term evolved into ‘anthocyanin’. In the early 20th century, Richard Willstätter demonstrated that these pigments are glycosides of flavylium‐based aglycones, which he termed anthocyanidins (Robinson 1935). From that point onwards, anthocyanins were no longer merely colours, but chemically defined metabolites.

After decades of classical biochemical and genetic research, the first sequences of genes underlying anthocyanin biosynthesis were identified and cloned (Martin *et al*. 1985; O'Reilly *et al*. 1985; Cone *et al*. 1986; Holton & Cornish 1995), establishing the anthocyanin biosynthesis pathway as one of the best‐understood specialized metabolic pathways in plants (Fig. 1). Today, a wide range of ecological and physiological functions is attributed to anthocyanins, including pollinator and seed disperser attraction (Davies *et al*. 2012; Renoult *et al*. 2014), sunscreen (Gould *et al*. 1995; Agati *et al*. 2021), camouflage (Givnish 1990) and mimicry (Lev‐Yadun & Inbar 2002). In biotechnology, anthocyanins are valued as strong antioxidants (Butelli *et al*. 2008, 2021) and food colourants (Appelhagen *et al*. 2018). Due to the visible phenotypes of mutations in the anthocyanin biosynthesis pathway, the pathway and its regulation have also been studied as a model system in evolutionary biology (Smith & Rausher 2011; Smith *et al*. 2013; Marin‐Recinos & Pucker 2024).

**Fig. 1:**
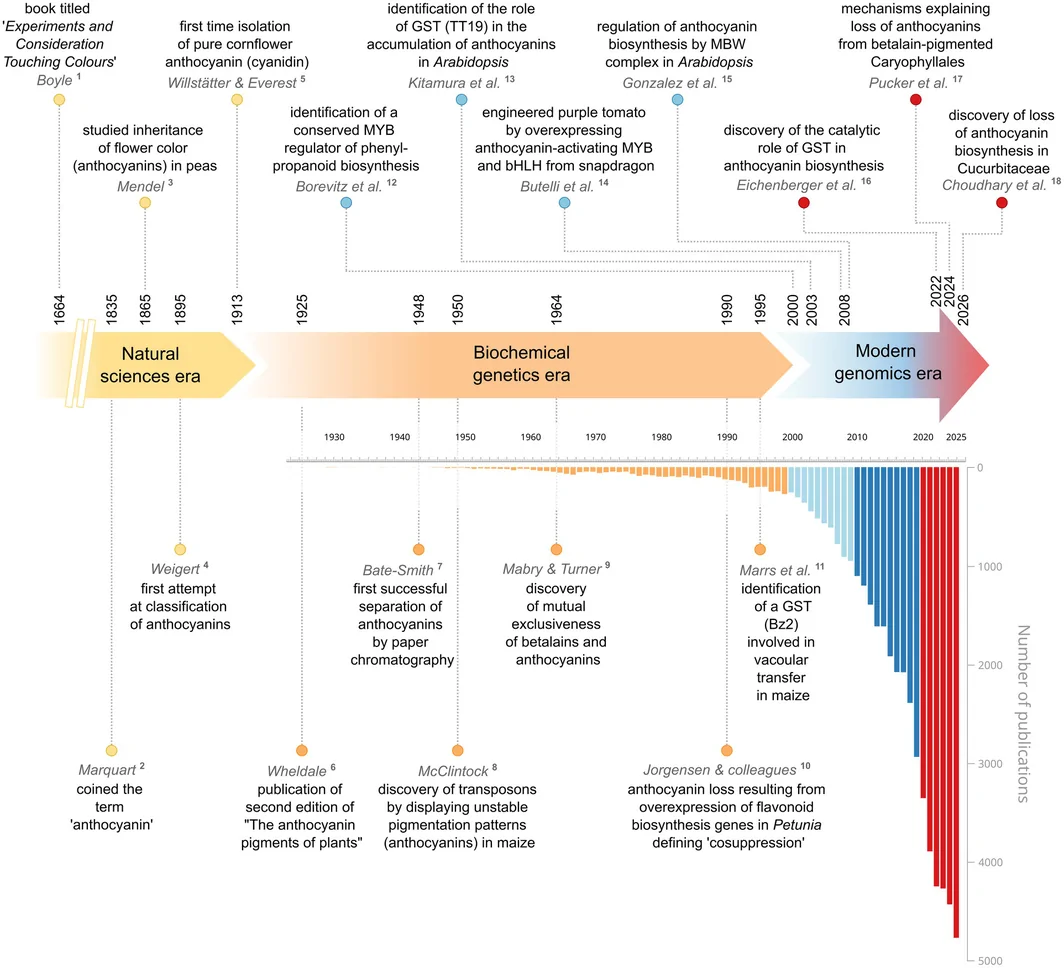
Timeline of fundamental discoveries and milestones in the anthocyanin biosynthesis research. The number of publications per year (from 1920 onwards) was retrieved from PubMed or Web of Science using the query ‘anthocyanin OR anthocyanidin OR leucoanthocyanidin OR cyanidin OR delphinidin OR pelargonidin OR malvidin OR petunidin OR peonidin’. References: 1 (Boyle 1664); 2 (Marquart 1835); 3 (Mendel 1865); 4 (Weigert 1895); 5 (Willstätter & Everest 1913); 6 (Wheldale Onslow 1925); 7 (Bate‐Smith 1948); 8 (McClintock 1950); 9 (Mabry & Turner 1964); 10 (Napoli *et al*. 1990; Jorgensen 1995); 11 (Marrs *et al*. 1995); 12 (Borevitz *et al*. 2000); 13 (Kitamura *et al*. 2004); 14 (Butelli *et al*. 2008); 15 (Gonzalez *et al*. 2008); 16 (Eichenberger *et al*. 2023); 17 (Pucker *et al*. 2024); 18 (Choudhary *et al*. 2026).

The anthocyanin biosynthesis pathway branches from the flavonoid biosynthesis (Winkel‐Shirley 2001; Grünig *et al*. 2025). Enzymes for the biosynthesis include chalcone synthase (CHS), chalcone isomerase (CHI), flavanone 3‐hydroxylase (F3H), dihydroflavonol 4‐reductase (DFR), anthocyanidin synthase (ANS), anthocyanin‐related glutathione S‐transferase (arGST), UDP‐dependent anthocyanidin 3‐O‐glucosyltransferase (U3GT) and additional modifying enzymes (Fig. 2a) (Grünig *et al*. 2025). Following F3H, the two optional enzymes flavonoid 3′‐hydroxylase (F3′H) and flavonoid 3′,5′‐hydroxylase (F3′5′H) can add one or two hydroxyl groups, respectively, which alter the biochemical properties of the intermediate, ultimately leading to different anthocyanin colours. The simplest pathway leads to orange pelargonidin derivatives; one additional hydroxylation yields pink cyanidin derivatives, and two additional hydroxylations yield blue delphinidin derivatives (Schwinn *et al*. 2014).

**Fig. 2:**
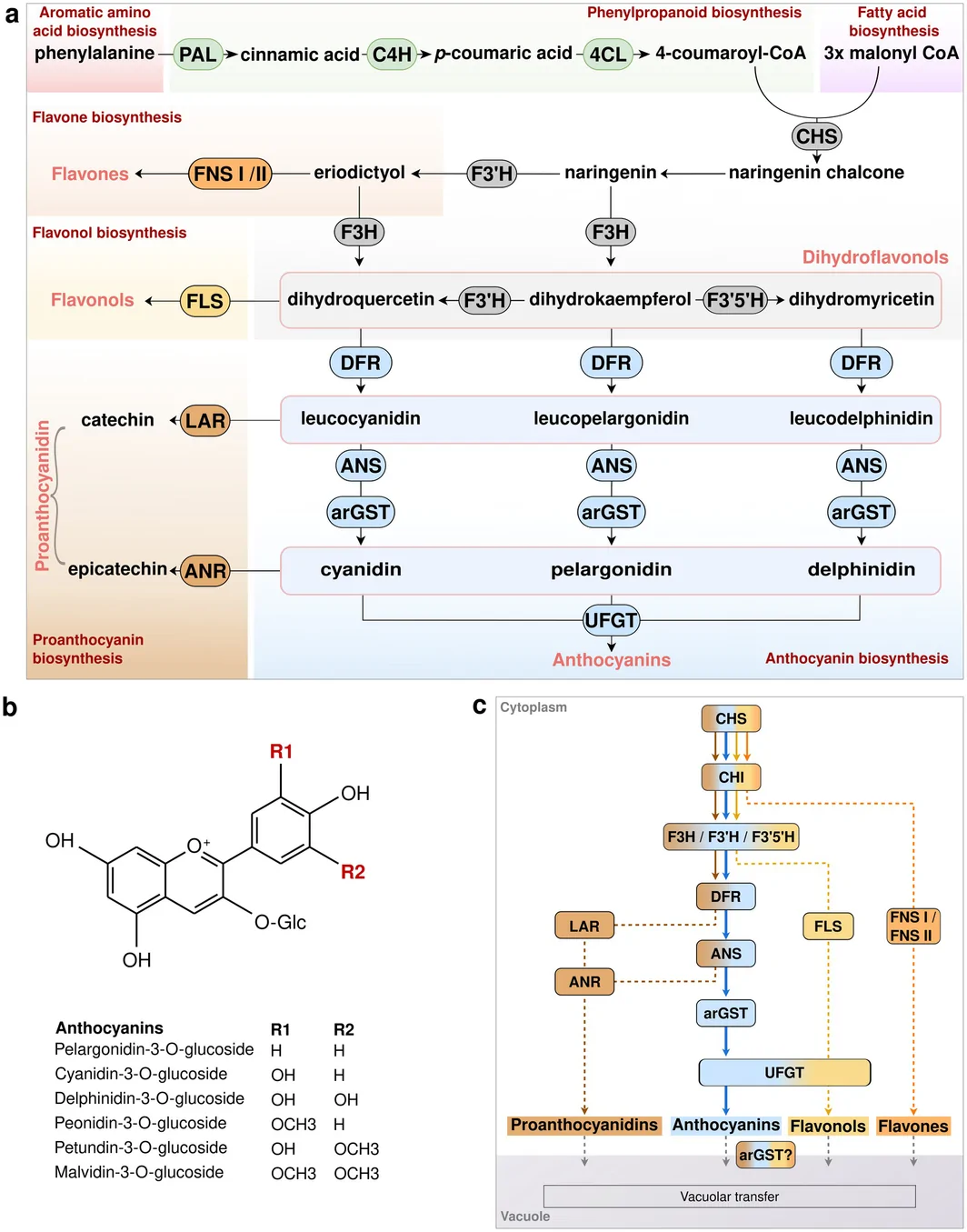
The core pathway for anthocyanin biosynthesis. (a) Simplified illustration of the flavonoid biosynthesis pathway in plants, highlighting the major branching pathways. The anthocyanin biosynthesis pathway and genes are shown in blue. Gene abbreviations not given in text: 4CL, 4‐coumarate‐CoA ligase; ANR, anthocyanidin reductase; C4H, cinnamate 4‐hydroxylase; FLS, flavonol synthase; FNS, flavone synthase; LAR, leucoanthocyanidin reductase; PAL, phenylalanine ammonia lyase. (b) Basic structure of an anthocyanin and representative anthocyanins. (c) Overlap of enzymes participating in the different branches of flavonoid biosynthesis. Flavone (orange), flavonol (yellow), anthocyanin (blue) and proanthocyanidin (brown) branches are shown.

The transcriptional activation of anthocyanin biosynthesis involves three transcription factors from different protein families that form a complex (MBW): MYB, bHLH and WD40 (Gonzalez *et al*. 2008; Grünig *et al*. 2025). The MYB component is considered the most specific, as it appears to be committed to anthocyanin biosynthesis (Wheeler *et al*. 2022; Marin‐Recinos & Pucker 2024). The bHLH and WD40 components have a range of functions in plant development and physiology beyond regulating anthocyanin biosynthesis. Several other transcriptional regulators have been reported (Grünig *et al*. 2025), including a number of transcriptional repressors (LaFountain & Yuan 2021).

In many species, the activating MYB proteins belong to the subgroup 6 (SG6) R2R3‐MYB family. Representative examples include ANTHOCYANIN2 in petunia (Quattrocchio *et al*. 1999) and the partially redundant MYB75 (PAP1), MYB90 (PAP2), MYB113 and MYB114 proteins in *Arabidopsis thaliana* (Stracke *et al*. 2001). Anthocyanin biosynthesis is further regulated by numerous endogenous and environmental signals. Light is one of the strongest inducers of anthocyanin accumulation (Nowak *et al*. 2024; Ye *et al*. 2026), while temperature (Xue *et al*. 2021), nutrient availability (Wang *et al*. 2023) and phytohormones such as jasmonic acid, ethylene, abscisic acid and sugars influence the activity of the regulatory network in a species‐ and tissue‐dependent manner (Wang *et al*. 2020, 2022; Zhao *et al*. 2025). Beyond transcriptional regulation, epigenetic mechanisms including DNA methylation, histone modifications and non‐coding RNAs have emerged as additional regulators that modulate chromatin accessibility and the expression of anthocyanin biosynthetic genes (Cai *et al*. 2019). Post‐translational regulation of MBW components, for example through phosphorylation and ubiquitin‐mediated protein turnover, further fine‐tunes anthocyanin production in response to developmental and environmental cues (Yang *et al*. 2022).

Despite decades of intensive study of anthocyanin biosynthesis as a model system, major discoveries have emerged in recent years. This includes arGST as an additional biosynthesis enzyme (Eichenberger *et al*. 2023) and the complete loss of anthocyanin biosynthesis in Cucurbitaceae (Choudhary *et al*. 2026). Patterns of duplication, subfunctionalization, loss and convergent evolution across angiosperms suggest that this seemingly ‘textbook’ pathway continues to yield unexpected insights.

Here, we revisit nearly two centuries of anthocyanin research to distinguish what is firmly established from what is inferred, assumed or still unknown. By highlighting overlooked observations, unresolved evolutionary questions and modern methodologies, we aim to outline a research agenda for the next phase of anthocyanin research.

## MAIN

### Is there a dedicated anthocyanin biosynthesis?

The set of genes reported as ‘anthocyanin biosynthesis’ varies considerably across studies. In some cases, the entire upstream core flavonoid pathway is implicitly treated as committed to anthocyanin biosynthesis, overlooking the multiple competing branches that lead to other end products. In others, DFR and ANS are identified as core components of the pathway, while additional steps such as arGST, glycosyltransferases and other modifying enzymes are often neglected.

The flavonoid biosynthesis is best understood as a highly complex interconnected network with branches leading to chemically distinct products, including flavones, flavonols, anthocyanins and proanthocyanidins (Winkel‐Shirley 2001; Grünig *et al*. 2025). The early steps are largely shared, and metabolic flux is directed into specific branches only at later steps. There is a substantial overlap in participating enzymes involved in anthocyanin and proanthocyanidin biosynthesis (Fig. 2b). Both pathways require DFR and ANS, indicating that these enzymes are not uniquely committed to anthocyanin biosynthesis. Recently, arGST was characterized as an enzyme in anthocyanin biosynthesis (Eichenberger *et al*. 2023), after a role in anthocyanin transport was postulated and widely propagated for over two decades (Mueller *et al*. 2000; Pucker & Selmar 2022). The *Arabidopsis thaliana* arGST (*transparent testa 19, tt19*) mutant was originally identified as defective in seed coat pigmentation, that is proanthocyanidin biosynthesis (Kitamura *et al*. 2004; Sun *et al*. 2012), which raises the question of whether this enzyme might be involved in both anthocyanin and proanthocyanidin biosynthesis. Plant lineages without arGST orthologs provide first hints for the dispensability of arGST in the context of proanthocyanidin biosynthesis and also offer an opportunity to explore this in greater detail (Pucker *et al*. 2024; Khatun *et al*. 2025). While grasses (Poaceae) possess Bz2 as a functional replacement of otherwise canonical arGSTs (TT19/An9 orthologues) (Khatun *et al*. 2025), several Caryophyllales families appear to lack a functional equivalent, but still harbour proanthocyanidin biosynthesis genes (Pucker *et al*. 2024). This suggests that arGST is dispensable for proanthocyanidin production–at least in some plant lineages. Meanwhile, the long‐standing model of arGST‐mediated anthocyanin transport has not been revisited in light of its discovery as an enzyme. Vesicle‐based anthocyanin transport mechanisms have also been postulated and supported by experimental evidence (Poustka *et al*. 2007; Pucker & Selmar 2022). Since functional redundancy is unlikely to be maintained over long evolutionary timescales, it seems improbable that two fully redundant transport systems coexist. In summary, these observations suggest that arGST might fulfil a more specific role as an enzyme in anthocyanin biosynthesis rather than acting as a general requirement for flavonoid biosynthesis. The step following arGST in anthocyanin biosynthesis is catalysed by a glycosyltransferase. In *A. thaliana*, UGT78D2 (At5g17050) not only converts unstable colourless anthocyanidins into anthocyanins, but also appears to modify flavonols (Tohge *et al*. 2005). In summary, these observations suggest that there might be no set of enzymes exclusively dedicated to anthocyanin biosynthesis. Instead, a coordinated activation of genes needed for the biosynthesis, combined with selective regulation and suppression of genes for competing branches, might form the anthocyanin biosynthesis. This would align with observations from evolutionary studies that reported the anthocyanin‐specific MYB activator as the most often implicated component in the anthocyanin biosynthesis block (Marin‐Recinos & Pucker 2024). While there are numerous enzymes for further modifications of anthocyanins (Grünig *et al*. 2025), these extend beyond the core biosynthetic steps. The multifunctionality observed in the anthocyanin biosynthesis genes aligns with the established role of enzyme promiscuity shaping the diversity of the (specialized) metabolism in plants (Leong & Last 2017; Yokoyama 2024). For practical reasons, however, it remains useful to define a minimal functional module for anthocyanin production. This core set, comprising the DFR, ANS, arGST and UFGT, enables the synthesis of stable anthocyanin pigments and provides a working definition of ‘anthocyanin biosynthesis’.

### Why is there independent evolution of certain steps in the anthocyanin biosynthesis?

Anthocyanin biosynthesis has generally been considered to be well conserved across land plants (Falcone Ferreyra *et al*. 2012; Xu *et al*. 2015; Tohge *et al*. 2017). Most documented exceptions involve lineage‐specific losses of anthocyanins, but these are often restricted to the species level (Marin‐Recinos & Pucker 2024). For a long time, the replacement of anthocyanins by betalains in several families of the Caryophyllales represented the only known scenario of large‐scale anthocyanin loss (Mabry 1964; Mabry & Turner 1964; Brockington *et al*. 2011; Timoneda *et al*. 2019). More recent studies, however, have covered additional cases of pathway gene loss and rewiring at the family level in Cucurbitaceae (Choudhary *et al*. 2026) and Poaceae (Khatun *et al*. 2025), respectively, suggesting a more dynamic evolutionary history of anthocyanin biosynthesis than previously assumed. However, most enzymes of the biosynthesis pathway appear to be monophyletic and broadly conserved across anthocyanin‐producing plant lineages.

An exception is the arGST encoding gene. The two classical arGSTs, Bz2 in maize (McLaughlin & Walbot 1987; Alfenito *et al*. 1998) and An9 in petunia (Alfenito *et al*. 1998), were discovered decades ago and share low sequence similarity between them. However, a reciprocal complementation experiment demonstrated that they apparently perform the same function in anthocyanin biosynthesis (Alfenito *et al*. 1998). Comprehensive phylogenetic analysis has shown that they are members of different GST lineages, with the Bz2 lineage being restricted to Poaceae, and the An9 lineage found in other anthocyanin‐pigmented plants (Fig. 3) (Khatun *et al*. 2025). It still remains an open question why the An9 lineage was lost in the grasses and why it was replaced by the Bz2 lineage. This might be associated with changes in the transcriptional regulation of anthocyanin biosynthesis in Poaceae.

**Fig. 3:**
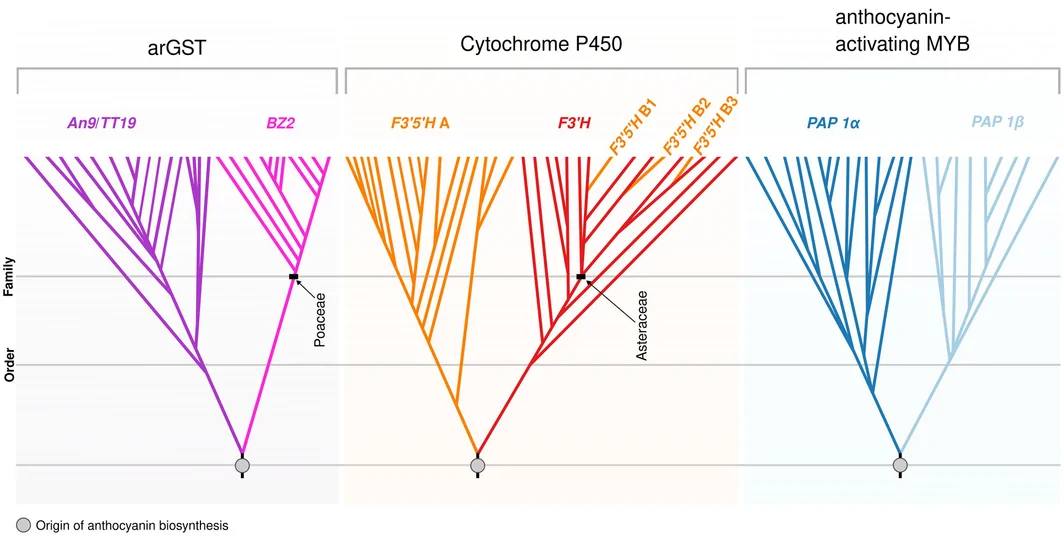
Simplified schematic illustration of some known duplication events affecting anthocyanin biosynthesis genes. From left to right: independent arGST lineages, independent F3′5′H evolution events and complex MYB relationships. An9, Anthocyanin9; Bz2, Bronze2; F3′5′H, flavonoid 3′,5′‐hydroxylase; F3′H, flavonoid 3′‐hydroxylase; PAP1α, Promoter of Anthocyanin Production 1α; PAP1β, Promoter of Anthocyanin Production 1β; TT19, Transparent Testa 19.

In general, the regulatory components of anthocyanin biosynthesis appear to be more evolutionarily flexible than the structural genes. In Poaceae, the canonical anthocyanin‐activating MYB lineage appears to have been lost and functionally replaced by a closely related MYB lineage (Khatun *et al*. 2025). It is possible that such a change in transcriptional regulation facilitated the recruitment of a different structural component, such as the distinct GST lineage. Broadly, a deep duplication event of the anthocyanin‐activating MYB in anthocyanin‐pigmented plants, followed by secondary loss of one MYB lineage in many plant lineages, has resulted in a complex evolutionary relationship between the anthocyanin regulators in different plants (Pucker *et al*. 2024). Therefore, insights from one species cannot always be directly transferred to other species if anthocyanin biosynthesis in these species is not controlled by an orthologous MYB.

The optional hydroxylation step of anthocyanidin precursors catalysed by F3′5′H is another well‐characterized example of independent evolution. F3′H (CYP75B) and F3′5′H (CYP75A) are sister lineages within the large cytochrome P450 family (Tanaka & Brugliera 2013). While both lineages split at the basis of anthocyanin‐pigmented plants (Tanaka & Brugliera 2013), there are several cases of independent evolution of F3′5′H activity from F3′H genes (Seitz *et al*. 2006, 2015; Lui *et al*. 2020). This repeated emergence is facilitated by the fact that only a small number of amino acid substitutions are required to shift enzyme specificity (Seitz *et al*. 2007). Therefore, it seems plausible that mutations at the required sites happened multiple times during evolution. Since the gene expression pattern of F3′H already aligns with other anthocyanin biosynthesis genes, derived gene copies would already show the gene activity needed to form anthocyanins.

In summary, the evolution of anthocyanin biosynthesis appears to be more complex than previously appreciated, including multiple events of gene loss, functional replacement and independent gene recruitment. Ecological factors such as shifts in pollinator or seed disperser preferences, adaptation to different light environments and responses to abiotic stresses may alter the selective pressures acting on pigment production and thereby promote changes in pathway composition. Likewise, changes in transcriptional regulation or the recruitment of alternative enzymes may allow the pathway to remain functional despite gene loss. Competition for shared metabolic precursors with other specialized metabolic pathways could provide an additional selective force favouring pathway rewiring. At present, however, these hypotheses remain largely untested, highlighting an important gap in our understanding of anthocyanin evolution.

### Does substrate hydroxylation pattern influence acceptance by ANS, arGST and downstream enzymes?

Dihydroflavonols, which differ in their B‐ring hydroxylation pattern, represent a central branching point in the flavonoid pathway. These intermediates can be utilized by flavonol synthase (FLS) and DFR to form flavonols or to form anthocyanins and proanthocyanidins, respectively. FLS and DFR show differential substrate preferences, which could serve as a mechanism to mitigate direct competition between these enzymes (Choudhary & Pucker 2024). For example, FLS in many species prefers the single hydroxylated substrate dihydrokaempferol, while DFR prefers the double hydroxylated substrate dihydroquercetin (Fig. 4). The activity of F3′H, which converts dihydrokaempferol into dihydroquercetin, therefore could be an important control point for directing metabolic flux between competing branches. A single amino acid residue in DFR has been reported as the decisive factor regarding substrate preference (Johnson *et al*. 2001). Based on this residue, which is position 133 in *A. thaliana*, the DFRs discovered in different plant species can be broadly classified into three types: the common DFR_N_ type, which mostly leads to cyaniding; the DFR_D_ type, which accepts double/triple hydroxylated substrates and can produce cyanidin/delphinidin; and the rarer DFR_A_ type, which favours single hydroxylated substrates and produces mostly pelargonidin (Johnson *et al*. 2001; Miosic *et al*. 2014).

**Fig. 4:**
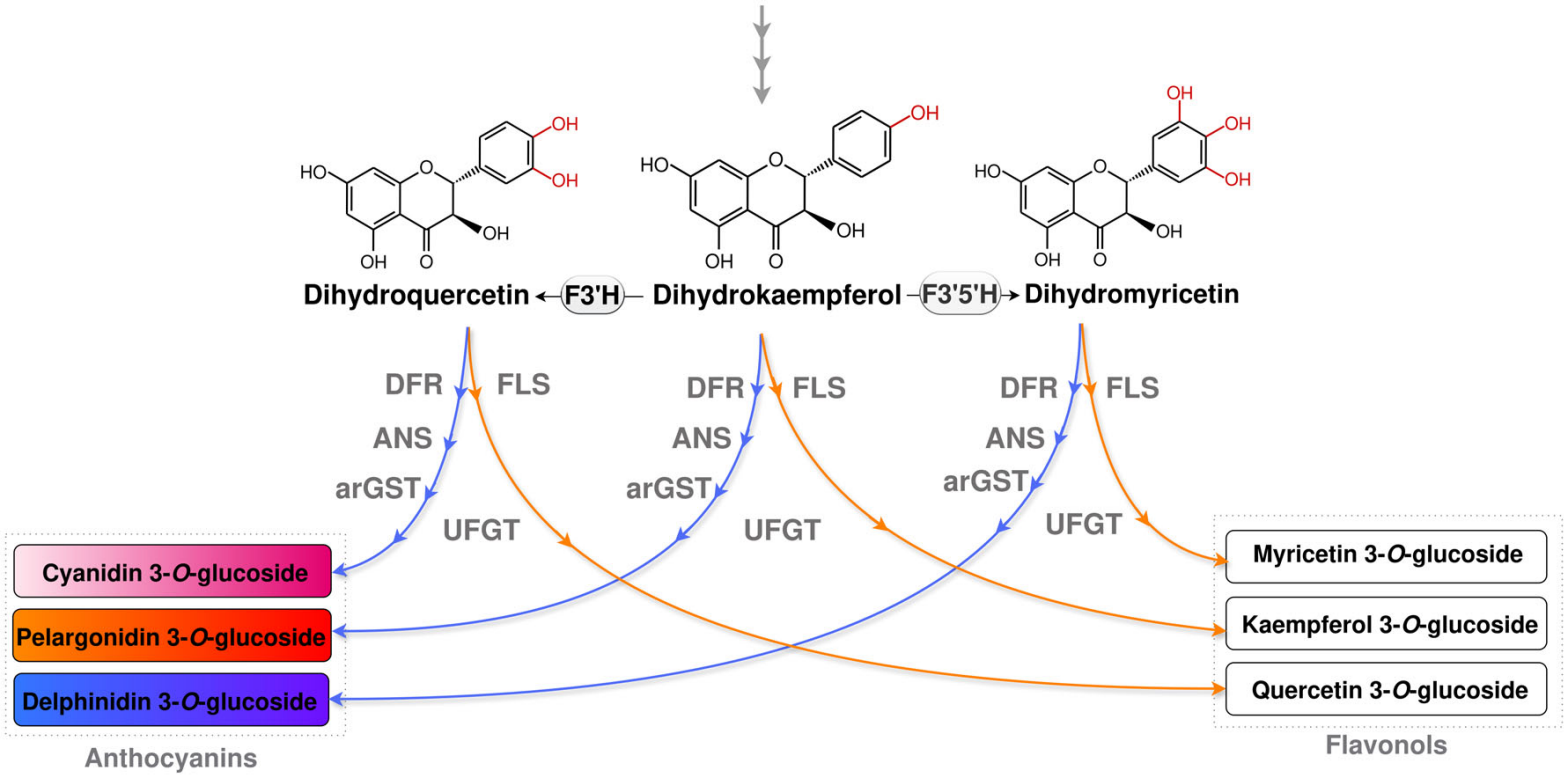
The major branching point in the flavonoid biosynthesis leading to representative anthocyanins (left) and flavonols (right). The hydroxylation pattern of dihydroflavonols is an important factor for the anthocyanin and competing flavonol biosynthesis. Dihydrokaempferol, with a single hydroxylation of the B ring, leads to orange pelargonidin derivatives; dihydroquercetin, with two hydroxylations at the B ring, results in pink cyanidin derivatives and dihydromyricetin, with three hydroxyl groups at the B ring, is converted into blue delphinidin derivatives. Different DFR types have preferences for substrates with a specific hydroxylation pattern. Competition between DFR and FLS is mitigated through differential substrate preferences and gene expression profiles.

These different substrates of DFRs result in the formation of leucoanthocyanidins with distinct hydroxylation patterns, which then serve as substrates for downstream enzymes. In the anthocyanin branch, these intermediates are further processed by ANS, arGST, glucosyltransferases and other decoration enzymes. This raises the question, ‘Do downstream enzymes in anthocyanin biosynthesis also exhibit substrate preferences linked to hydroxylation patterns?’ If ANS, arGST, or UFGTs show similar substrate‐specificity constraints as DFR, substrate hydroxylation could be the unifying principle governing pathway flux and product composition. Identifying such key residues, if any, would not only deepen our understanding of the pathway but could also provide opportunities in crop improvement and for the horticulture industry.

### Which factors control transcription of *arGST* , *F3* ′*H* and *F3* ′*5*′*H*?

Transcriptional regulation of anthocyanin biosynthesis appears to be the most flexible element and the most important factor during evolutionary change. Although the regulation of anthocyanin biosynthesis through the MBW complex is considered a model system for transcriptional control in eukaryotes, there are still significant knowledge gaps in plain sight. The MBW complex is a central component, but numerous additional factors have been reported that might fine‐tune the activation of different anthocyanin biosynthesis genes under specific conditions (Jaakola 2013; Liu *et al*. 2019; LaFountain & Yuan 2021; Grünig *et al*. 2025). Comparing the transcriptional regulation of arGSTs in the Bz2 and An9 lineage could help to understand fundamental differences, which might have provided an evolutionary advantage that facilitated the replacement of An9 by Bz2 in the Poaceae. Simultaneously, this would also contribute to answering the question of whether arGST is a committed player in anthocyanin biosynthesis.

Several hints suggest that the F3′H and F3′5′H encoding genes are not strongly coexpressed with other genes of the anthocyanin biosynthesis. For example, flavonol regulators of the MYB subgroup 7 lineage triggering *CHS*, *CHI* and *F3H* do not seem to activate *F3*′*H* in *A. thaliana* (Stracke *et al*. 2007). In our experience, the coexpression of *F3*′*H* with *DFR* and *ANS* in *A. thaliana* is rather weak. A precise regulatory control of *F3*′*H* and *F3*′*5*′*H* might be a mechanism to mediate the flux into the competing pathways of flavonol and anthocyanin biosynthesis (Choudhary & Pucker 2024).

### Is arGST only an enzyme or also involved in anthocyanin transport?

The evidence for the arGST role as an enzyme in anthocyanin biosynthesis (Eichenberger *et al*. 2023) questions the previous hypothesis that the protein would function in anthocyanin transport (Mueller *et al*. 2000). Originally, there was enzymatic activity reported for Bz2 (Marrs *et al*. 1995), but numerous subsequent studies explained the association of this protein with anthocyanin metabolism with a non‐enzymatic role as a ‘ligandin’ protecting anthocyanins during transport to the central vacuole (Mueller *et al*. 2000; Kitamura *et al*. 2004; Pucker & Selmar 2022). While there is no clear evidence for an arGST role in anthocyanin transport, changing this well‐established narrative is difficult. Ultimately, it is impossible to completely rule out a function in anthocyanin transport, but gathering evidence for a purely enzymatic role would be an important contribution to an accurate picture of anthocyanin metabolism.

### Do anthocyanins have an indispensable function?

Anthocyanins are best known for their role as visible pigments, contributing red to blue to purple coloration to plants. This pigmentation is directly linked to well‐established ecological functions, including the attraction of pollinators and seed dispersers (Winkel‐Shirley 2001; Grünig *et al*. 2025), as well as roles in camouflage and mimicry (Givnish 1990; Lev‐Yadun & Inbar 2002). These functions provide a clear explanation for the widespread occurrence of anthocyanins in angiosperms, that is, the flowering plants. However, a broader range of physiological roles, beyond pigmentation, has been attributed to anthocyanins, including photoprotection (Gould *et al*. 1995; Agati *et al*. 2021), antioxidant activity (Butelli *et al*. 2008) and tolerance to abiotic stresses (Chalker‐Scott 1999; Pietrini *et al*. 2002). Many of these functions are also described for other flavonoid classes from the branches of the flavonoid pathway. This raises the question whether these roles are specific to anthocyanins or instead properties of flavonoids as a class. From an evolutionary perspective, this distinction is particularly relevant. The core flavonoid pathway, including flavone and flavonol biosynthesis, is deeply conserved and likely originated early in land plant evolution (Davies *et al*. 2024). The complete suite of enzymes required for canonical anthocyanin biosynthesis appears to have originated first in gymnosperms (Piatkowski *et al*. 2020) and is most consistently observed in angiosperms, suggesting it might have evolved as a reproductive strategy. Large‐scale anthocyanin loss provides further insights. In several lineages of the Caryophyllales, betalains have replaced anthocyanins, resulting in the well‐known mutual exclusion of these two pigment systems (Timoneda *et al*. 2019). Comparative genomic studies have shown that betalain‐producing species have lost arGSTs and show loss‐of‐original‐function variants in anthocyanin‐activating MYB transcription factors, while harbouring an independently evolved betalain biosynthetic pathway (Sheehan *et al*. 2020; Pucker *et al*. 2024). Similarly, members of the Cucurbitaceae have lost several core anthocyanin biosynthesis genes, including DFR, ANS, arGST and anthocyanin‐activating MYBs, whereas carotenoids represent the predominant pigments responsible for fruit coloration (Choudhary *et al*. 2026). Together, Caryophyllales and Cucurbitaceae demonstrate that anthocyanins are not universally required for pigmentation and that alternative pigment systems can take over this role. Whether the broader physiological functions often attributed to anthocyanins, such as photoprotection or stress tolerance, are similarly replaceable remains an open question. If these functions were indispensable, large‐scale losses of the anthocyanin pathway would have been difficult to reconcile over large evolutionary times. In this context, anthocyanins may represent one of the interchangeable solutions from the flavonoid pathway rather than a necessarily required component.

### Promising methods for future discoveries

With the continued expansion of genomic and transcriptomic resources (Leebens‐Mack *et al*. 2019), the discovery of plant specialized biosynthesis pathways has accelerated considerably. Pathways that were once thought to be largely resolved are now being revisited, with new insights emerging from large‐scale datasets (Nützmann *et al*. 2016; Luo *et al*. 2026). Anthocyanin biosynthesis is a clear example of this trend, where recent findings have been enabled not by classical biochemistry alone, but by the increasing availability of high‐quality genome assemblies and expression data (Khatun *et al*. 2025; Choudhary *et al*. 2026).

Genome assemblies provide access to the full repertoire of genes within a species, while transcriptomic data offer complementary information on gene activity across tissues, developmental stages and environmental conditions. The combination of these resources has facilitated the application of co‐expression network analysis, synteny‐based comparisons and phylogenetic inference. Such strategies are particularly valuable for uncovering lineage‐specific genes or previously uncharacterized factors that may contribute to anthocyanin biosynthesis.

As has been the case for other areas of plant metabolism, the choice of study system strongly influences the types of insights that can be obtained (Dixon & Dickinson 2024). Classical model species such as petunia, maize and snapdragon have played a central role in establishing the genetic and biochemical framework of anthocyanin biosynthesis (Goodrich *et al*. 1992; Holton & Cornish 1995). However, these systems do not capture the full diversity of pathway organization observed across plant lineages (Khusnutdinov *et al*. 2021; Yu *et al*. 2023).

The Poaceae, for example, differ markedly from canonical systems in that they lack the typical anthocyanin‐activating MYB transcription factors and associated arGSTs. Instead, alternative regulators, including distinct MYB variants and the arGST protein Bz2, appear to fulfil analogous functions (Marrs *et al*. 1995; Alfenito *et al*. 1998; Eichenberger *et al*. 2023; Khatun *et al*. 2025). This pattern has been interpreted as evidence for a loss of canonical components followed by the re‐establishment of anthocyanin biosynthesis through non‐canonical elements (Khatun *et al*. 2025). Such evolutionary transitions raise the possibility that additional genes were lost, replaced or repurposed during this process, making the Poaceae a useful system for investigating the flexibility and rewiring of the anthocyanin pathway.

In contrast, members of the Cucurbitaceae appear to lack the core anthocyanin biosynthetic genes as well as their associated regulatory factors (Choudhary *et al*. 2026). This absence positions Cucurbitaceae as a natural system for studying anthocyanin biosynthesis. Comparative analyses across species may therefore highlight genes that are consistently associated with anthocyanin biosynthesis but are specifically absent in Cucurbitaceae, providing strong candidates for functional characterization. These candidates can subsequently be evaluated using established experimental approaches, including virus‐induced gene silencing, genome editing and protein interaction assays.

With the continued development of integrative genomic tools and functional validation techniques, the primary limitation is shifting from data generation to experimental accessibility across diverse plant systems. As a result, even well‐characterized pathways such as anthocyanin biosynthesis remain open to further discovery, particularly when examined across a broader evolutionary context.

## CONCLUSION

Recent studies suggest that there is still potential for fundamental discoveries in anthocyanin biosynthesis. The picture of a well‐conserved pathway is challenged by species‐specific differences and multiple cases of anthocyanin loss that have been revealed in recent years. The importance of transcriptional regulation emphasizes questions about the fine‐tuning of different steps and control mechanisms that can prevent competition between branches of the flavonoid biosynthesis. All these findings support anthocyanin biosynthesis as an excellent model system, as it also reflects the complexity, diversity and evolutionary flexibility of the specialized plant metabolism.

## AUTHOR CONTRIBUTIONS

BP designed the project and supervised the work. NC and BP wrote the manuscript. NK and NC designed figures. NC, NK and BP revised the manuscript.

## CONFLICT OF INTEREST STATEMENT

The authors declare that they have no competing interests.

## Data Availability

Data sharing not applicable to this article as no datasets were generated or analysed during the current study.
